# Guideline adherence and implementation of tumor board therapy recommendations for patients with gastrointestinal cancer

**DOI:** 10.1007/s00432-022-03991-6

**Published:** 2022-04-08

**Authors:** Alina Krause, Gertraud Stocker, Ines Gockel, Daniel Seehofer, Albrecht Hoffmeister, Hendrik Bläker, Timm Denecke, Regine Kluge, Florian Lordick, Maren Knödler

**Affiliations:** 1 Klinik und Poliklinik für Onkologie, Gastroenterologie, Hepatologie, Pneumologie und Infektiologie, Universitätsklinikum Leipzig, Leipzig, Germany; 2 Klinik und Poliklinik für Viszeral-, Transplantations-, Thorax- und Gefäßchirurgie, Universitätsklinikum Leipzig, Leipzig, Germany; 3 Institut für Pathologie, Universitätsklinikum Leipzig, Leipzig, Germany; 4 Klinik und Poliklinik für Diagnostische und Interventionelle Radiologie, Universitätsklinikum Leipzig, Leipzig, Germany; 5 Klinik und Poliklinik für Nuklearmedizin, Universitätsklinikum Leipzig, Leipzig, Germany; 6 Universitätsklinikum Leipzig, Universitäres Krebszentrum Leipzig (UCCL), Leipzig, Germany

**Keywords:** Adherence, Guidelines, Multidisciplinary tumor board, Gastrointestinal tumors, Implementation

## Abstract

**Purpose:**

Although participation in multidisciplinary tumor boards (MTBs) is an obligatory quality criterion for certification, there is scarce evidence, whether MTB recommendations are consistent with consensus guidelines and whether they are followed in clinical practice. Reasons of guideline and tumor board deviations are poorly understood so far.

**Methods:**

MTB’s recommendations from the weekly MTB for gastrointestinal cancers at the University Cancer Center Leipzig/Germany (UCCL) in 2020 were analyzed for their adherence to therapy recommendations as stated in National German guidelines and implementation within an observation period of 3 months. To assess adherence, an objective classification system was developed assigning a degree of guideline and tumor board adherence to each MTB case. For cases with deviations, underlying causes and influencing factors were investigated and categorized.

**Results:**

76% of MTBs were fully adherent to guidelines, with 16% showing deviations, mainly due to study inclusions and patient comorbidities. Guideline adherence in 8% of case discussions could not be determined, especially because there was no underlying guideline recommendation for the specific topic. Full implementation of the MTBs treatment recommendation occurred in 64% of all cases, while 21% showed deviations with primarily reasons of comorbidities and differing patient wishes. Significantly lower guideline and tumor board adherences were demonstrated in patients with reduced performance status (ECOG-PS ≥ 2) and for palliative intended therapy (*p* = 0.002/0.007).

**Conclusions:**

The assessment of guideline deviations and adherence to MTB decisions by a systematic and objective quality assessment tool could become a meaningful quality criterion for cancer centers in Germany.

**Supplementary Information:**

The online version contains supplementary material available at 10.1007/s00432-022-03991-6.

## Introduction

Over the years, case discussions in multidisciplinary tumor boards (MTBs) have emerged as standard for oncological treatment planning in Germany and worldwide. In MTBs, physician experts meet regularly to discuss therapy options for the patients presented there, considering patient- and tumor-specific characteristics. The task of the MTB is to formulate therapy recommendations that are based on scientific evidence on the one hand and individually adapted to each patient on the other hand. Due to the increasing complexity and fast-moving nature of oncological treatment options, professional cooperation between several disciplines is required (El Saghir et al. 2014; Hollunder et al. 2018), with the aim of providing the best possible medical care for oncological patients.

Numerous studies have already shown that oncological patients benefit from multidisciplinary approach in health care (Davies et al. 2006; Freeman et al. 2011; Freytag et al. 2020; Hsu et al. 2016; Huang et al. 2021; Prades et al. 2015). They benefit from a guideline-compliant and, thus, scientifically proven therapy (Jaap et al. 2018; Thiels et al. 2020; Visser et al. 2012; Worhunsky et al. 2015; Zhao et al. 2018), and further from the actual implementation of the therapy recommended in MTBs (Blay et al. 2017; Palmer et al. 2011; Stephens et al. 2006; Visser et al. 2012).

The proven benefits for patients should give enough reasons for further development of qualified MTBs (Hollunder et al. 2018). Therefore, guideline adherence of tumor board recommendations and tumor board adherence in the course of treatment are of great importance. Understanding the reasons for non-adherence to guidelines and MTB recommendations could improve quality of care as well as the understanding of potential influences from patient- and tumor-specific factors. Therefore, the applicability of oncology guidelines requires closer consideration and analysis.

According to the National program of certified oncology centers in Germany, MTBs are periodically reviewed by expert commissions for their regularity and coordinated infrastructure (Kowalski et al. 2017). However, systematic quality control regarding guideline adherence and consistent implementation of tumor board recommendations into clinical care of patients has been pending so far. Causes of guideline deviations or deviating implementation of MTB therapy recommendations have neither been sufficiently researched nor systematically surveyed. One potential reason for this is the lack of a uniform and, above all, transparent definition of “adherence” or “deviation” for analysis (Hollunder et al. 2018; Niño de Guzmán et al. 2020). Due to the use of non-objective measurement instruments, a systematic comparison of previous study results is not promising.

For the here presented study, we, therefore, developed a transparent and objective quality assessment model for determining and graduating adherence and used it to investigate guideline and tumor board adherence in MTBs for gastrointestinal cancers. We focused on the causes of their deviations and on the possible impact of specific patient and tumor characteristics.

## Methods

In this single center study, adherence testing was performed in the weekly MTB for gastrointestinal cancers of the University Hospital Leipzig (UKL)/University Cancer Center Leipzig (UCCL), Germany. Retrospectively, therapy recommendations were assessed for their adherence to the corresponding national treatment guidelines (= guideline adherence) and for their implementation (= tumor board adherence) for each patient case, which was presented between January 2020 and December 2020. For MTB cases showing deviations of guideline or tumor board recommendations, the underlying causes and possible influencing factors of patients and tumor disease were explored.

### Gastrointestinal MTB

At the UKL/UCCL, the gastrointestinal MTB takes place on a weekly basis. All patients with malignant gastrointestinal tumor diseases are discussed in this tumor board, except for hepatocellular carcinoma patients, for whom an extra tumor board exists. Each MTB consists of at least one representative of each subject area, which is involved in the patient’s treatment. Typical members according to institutional standard operating procedures are: specialists in surgery, medical oncology, gastroenterology, radiotherapy, pathology, radiology, nuclear medicine and psychooncology. These specialists meet for a panel concerning the treatment plan for patients, which were announced before. Up to 30–40 patient cases are analyzed weekly during a time frame of 2 h. Treatment recommendations are recorded electronically in a specially established and standardized protocol within the individual patient’s electronic chart in the clinic information system (SAP).

### Patient cohort

Patient cases were eligible for enrolment, if they met the following selection criteria: presence of a malignant tumor of the gastrointestinal tract and discussion of a disease status in the MTB with consequent treatment recommendation. Tumor boards of patients with benign tumors or precancerous lesions were excluded from this study. Furthermore, minors, pregnant and breastfeeding women, and patients unable to give consent were excluded (Fig. 2).

### Treatment guidelines

The guideline adherence of the tumor boards was determined based on tumor-specific German AWMF (the association of the scientific medical societies in Germany; AWMF = “Arbeitsgemeinschaft der Wissenschaftlichen Medizinischen Fachgesellschaften e.V.”; www.awmf.org) and Onkopedia (diagnostic and treatment guidelines of the German Society of Hematology and Oncology (DGHO = “Deutsche Gesellschaft für Hämatologie und Medizinische Onkologie e.V.”); www.onkopedia.com) therapy guidelines. These guidelines are standardized, regularly updated and have the highest status on a national level in Germany.

At the time of study evaluation, valid guidelines of the AWMF were available for the following carcinomas: esophageal cancer, esophagogastric junction (EGJ) tumors and gastric cancer, neuroendocrine tumors, colorectal cancer and anal cancer (since December 2020). For pancreatic cancer, biliary tumors and gastrointestinal stromal tumors (GIST), no current AWMF guidelines were available in 2020. Therefore, associated Onkopedia guidelines were used for these tumor entities. In addition, therapy recommendations from the German AWMF guideline for “Palliative Care for Patients with Incurable Cancer” were included into adherence testing.

### Data sources

All patient data were collected from the MTB protocols using a structured case report form (CRF). The implementation of the MTBs therapy recommendations was tracked via the hospital’s internal digital patient file over a time period of 3 months. For patients who received their following treatment outside the University Hospital of Leipzig, we used the data collection of the UCCL network and of the Clinical Cancer Registry of Saxony (KKR = “Klinisches Krebs-Register” Sachsen).

### Systematic adherence assessment

**Fig. 1:**
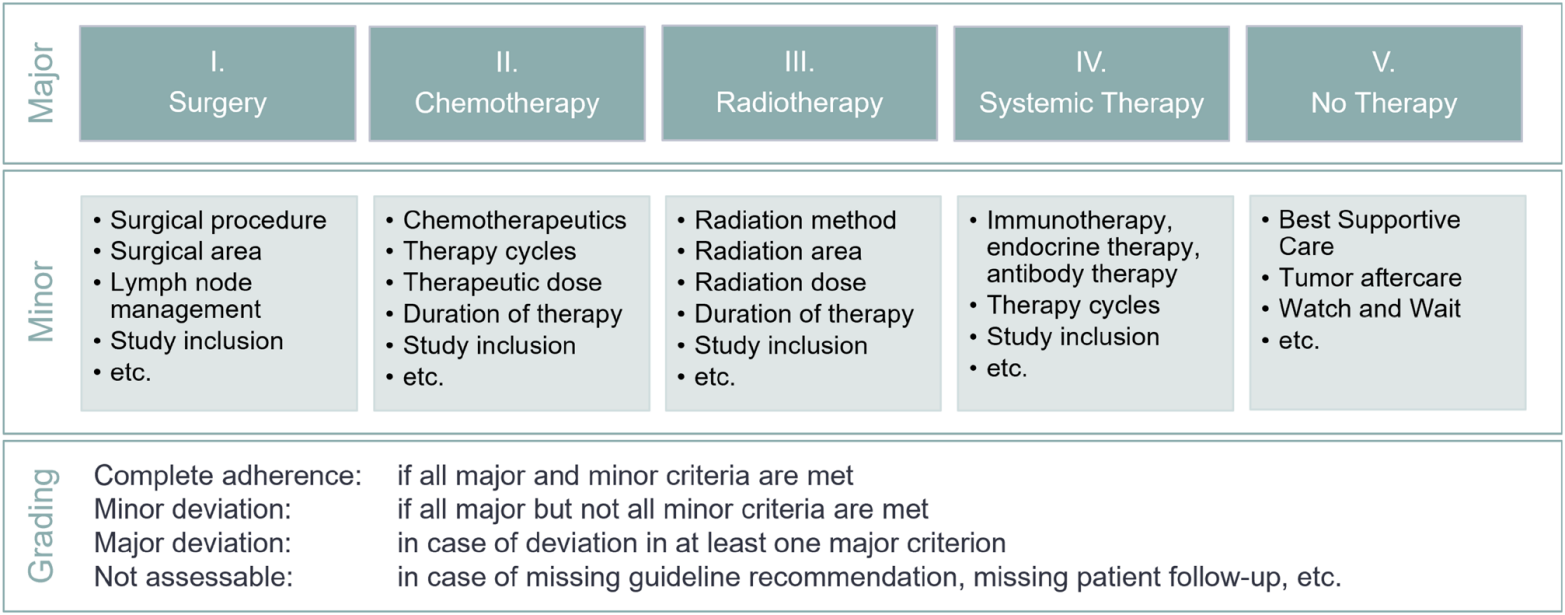
Adherence classification model with Major and Minor criteria

To perform an objective and systematic adherence assessment, a classification model for graduated assessment of guideline and tumor board adherence was developed. This model consists of predefined major and minor criteria (Fig. 1). The major criteria are based on the different treatment modalities in oncology. Minor criteria include therapy details of the associated major criteria. According to this classification model, “complete adherence” corresponds to concordance in all major and minor criteria between the tumor board recommendation and the associated guideline recommendation and the patient’s course of therapy. A “minor deviation” exists when there is concordance between all major criteria but not all minor criteria. When there is a deviation in at least one major criterion, we refer to this as a “major deviation”. In case guideline or tumor board adherence cannot be determined, the respective tumor board recommendations correspond to the fourth category “non-assessable adherence”.

### Causes of deviation

For cases showing deviations from guidelines or MTB recommendations, the underlying causes of deviation were collected and categorized based on available patient documentation data. Possible causes of guideline deviations include deviating physician recommendations, patient requests, comorbidities, study inclusion of patients, organizational reasons (e. g. availability of therapies, accessibility of therapy sites, etc.), and insufficient guideline topicality. Causes of tumor board deviations were differing physician recommendations, patient requests, comorbidities and organizational reasons, but also therapy complications, patient’s death, tumor factors (e.g., increase in the extent of tumor disease, novel molecular findings, response to therapy), and the initiation of therapy after the follow-up period of 3 months.

Two exemplary MTB cases with adherence assessment and causes of deviation are attached in a supplemental table (Supplemental Table 1).

### Statistical analyses

Patient characteristics, guideline and tumor board adherence, as well as reasons for their deviations, were determined using descriptive statistics. To detect possible statistically significant associations of patient and tumor factors with guideline or tumor board adherence, first, chi-square tests for independence and Fisher’s exact tests were performed. Second, two multivariate ordinal logistic regression models were constructed to ascertain the independent association of these factors with adherence to guidelines and tumor board recommendations. For statistical analysis of our data set, we used IBM SPSS Statistics for Windows version 27.0 and considered a *P* value of < 0.05 (two-tailed) to be statistically significant.

### Data monitoring and review workflow 

Eligibility assessment, adherence assessment and risk of bias assessment were conducted by one author (AK) and cross-checked by a second author (MK). For outstanding questions and unclear assessments, a third author (FL) was regularly consulted for a further cross-check and final evaluation (6-eyes principle). The assignment of the respective causes of deviation was always performed jointly by two authors or, in case of ambiguity, by consulting a third author, analogously to the determination of adherence.

## Results

**Fig. 2:**
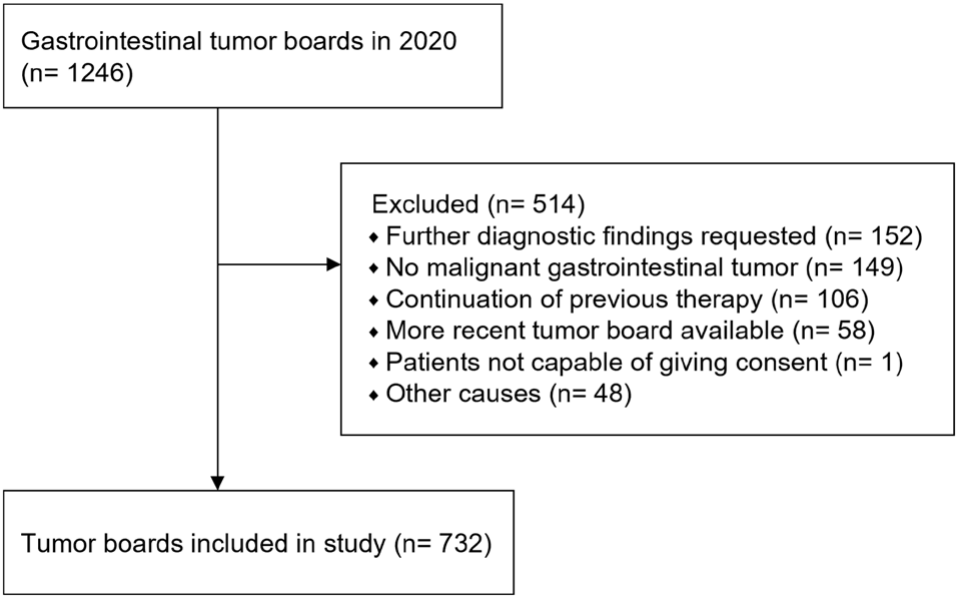
Study profile

In total, 1246 gastrointestinal MTB case discussions took place at the UKL/UCCL between January 1st and December 31st, 2020. Of these, 732 MTB cases were assigned to the study according to the inclusion criteria (see Fig. 2). Patient and tumor characteristics are summarized in Table 1.

**Table 1:** Patient demographics and tumor characteristics

Patient characteristics	*n*	%	Tumor characteristics	*n*	%
Tumor boards	732	100			
Patients	470	100	Tumor entities		
Sex			Colorectal cancer	283	38.7
Male	501	68.4	EGJ-tumors/gastric cancer	180	24.6
Female	231	31.6	Esophageal cancer	84	11.5
Age			Pancreatic cancer	68	9.3
Mean/minimum–maximum	62.91/20–99		Biliary tract tumors	46	6.3
25th percentile	55		Neuroendocrine tumors	31	4.2
50th percentile	64		Anal cancer	14	1.9
75th percentile	72		GIST	12	1.6
ECOG performance status			Pseudomyxoma peritonei	12	1.6
ECOG 0	326	62.8	Small intestine cancer	2	0.3
ECOG 1	150	28.9	UICC stage		
ECOG 2	30	5.8	UICC-Stage I	54	8.3
ECOG 3	11	2.1	UICC-Stage II	59	9.1
ECOG 4	2	0.4	UICC-Stage III	169	26.0
ECOG not available	213	–	UICC-Stage IV	367	56.5
Medication			UICC not available	83	–
Mean/minimum–maximum	4.69/0–18		Therapy intention		
< 5 medications per day	354	51.5	Curative	342	47.0
≥ 5 medications per day	334	48.5	Palliative	386	53.0
Medication not available	44	–	Therapy intention not available	4	–

Anti-tumoral therapies were recommended for 11 gastrointestinal cancer entities, with 75% of the MTB cases involving patients with colorectal cancer and esophagogastric cancer, respectively. 53% of the MTB recommendations were about therapies in palliative intent. The recommended anti-tumoral therapies are shown in Table 2. Minor criteria (therapy details) regarding these treatment modalities were reported in 44% of the evaluated MTB cases.

**Table 2:** Tumor board adherence to treatment modalities

Treatment modalities	Recommended in MTB, *n* (%)	Fully implemented, *n* (%)	Deviation from MTB, *n* (%)	Non-assessable implementation, *n* (%)
Surgery	196 (21.9)	170 (85.9)	24 (12.1)^a^	4 (2.0)
Chemotherapy	401 (44.9)	216 (53.9)	114 (28.4)	71 (17.7)
Radiotherapy	104 (11.6)	80 (76.9)	19 (18.3)	5 (4.8)
Systemic therapy	82 (9.2)	53 (64.6)	19 (23.2)	10 (12.2)
No tumor-directed therapy	107 (12.0)	77 (72.0)	1 (0.9)	29 (27.1)

^a^2 additional surgeries without associated tumor board recommendations

### Guideline adherence

76% (*n* = 557) of the total 732 MTB recommendations were fully adherent to the associated treatment guidelines. MTBs of patients with neuroendocrine tumors and gastrointestinal stroma tumors (GIST) had the highest rate of guideline adherence (97% and 92%), while in contrast the highest rates of guideline deviations occurred in EGJ-tumors/gastric carcinomas (23%), esophageal carcinomas (21%), and in biliary tumors (17%).

16% (*n* = 115) of MTB recommendations showed deviations from guidelines, including 9% minor and 7% major deviations by definition. The main causes of guideline deviations were study inclusion of patients (51%), comorbidities (24%), and physician recommendation deviating from the guidelines (18%). A deviating patient request and a lack of guideline topicality (3% each) were only rare causes in the gastrointestinal MTB.

Guideline adherence could not be assessed in a total of 8% (*n* = 60) of MTB recommendations. The main reason for this was the lack of guideline recommendations for the respective tumor board cases (80%).

**Fig. 3:**
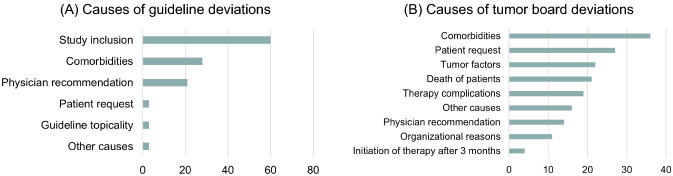
Causes of guideline (**A**) and tumor board (**B**) deviations

At the time of study evaluation, German guideline recommendations were missing for the following tumor entities: pseudomyxoma peritonei/low-grade appendiceal mucinous neoplasm (LAMN), small bowel cancer and anal cancer (until December 2020). In addition, guideline recommendations were lacking for palliative third- and fourth-line chemotherapy and for symptomatic local therapies of distant metastases in tumor patients who were comorbid and ineligible for chemotherapy. In general, guideline adherence could more frequently not be assessed in case of palliative compared to curative MTB recommendations. Further causes of non-assessable guideline adherence were non-guideline-compliant pre-therapy of patients (12%) and unclear tumor board recommendations (8%) (Fig. 3).

### Tumor board adherence

Complete tumor board adherence was demonstrated by 64% (*n* = 469) of all MTB recommendations. In 15% (*n* = 112), it was not possible to determine tumor board adherence due to the lack of patient follow-up. 21% (*n* = 151) of all therapies deviated from the original tumor board recommendations, 8% with minor, and 12% with major deviations. Main reasons of MTB deviations were comorbidities (21%) and deviating patient requests (16%). Patients with GIST, anal carcinoma and colorectal carcinoma had the highest tumor board adherence (83%, 71%, and 69%), whereas the highest rates of deviation occurred in patients with pseudomyxoma peritonei (33%), biliary tract tumors (22%), and esophageal cancer (20%).

**Fig. 4:**
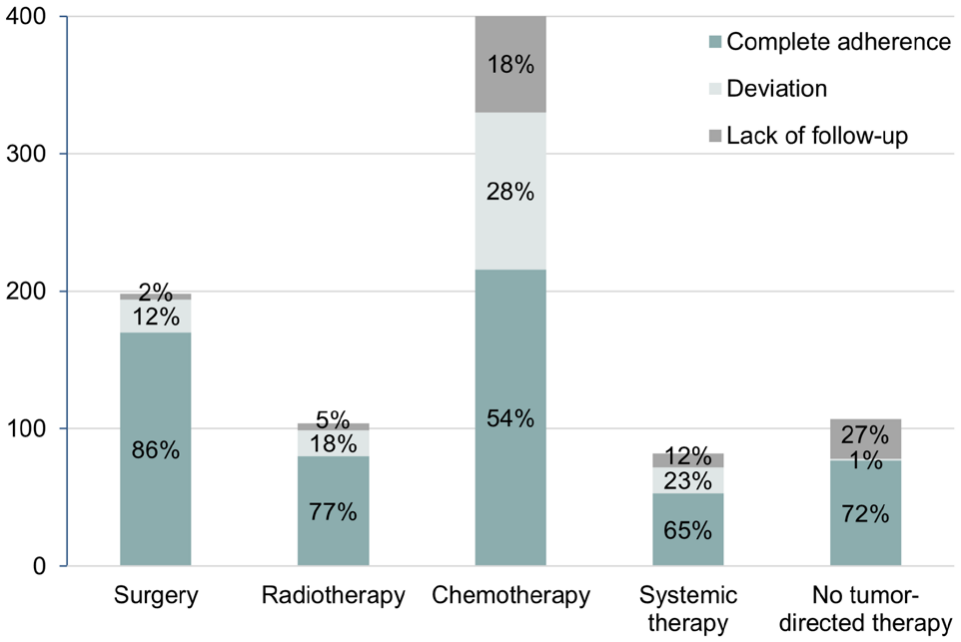
Tumor board adherence to treatment modalities

In regards of treatment modalities, the highest tumor board adherence was seen with surgery at 86%. In contrast, only 54% of chemotherapies were fully implemented as recommended by the MTB, and 28% deviated during the course of therapy. The lowest tumor board deviation rate of 1% was recorded for the recommendation to refrain from tumor-directed therapy (Fig. 4 and Table 2).

### Impact of patient characteristics

After controlling for patient and tumor factors on multivariate ordinal logistic regression analysis, no statistically significant dependence of guideline and tumor board adherence on patient age, gender and daily number of medications were found (*P* > 0.05). Three factors were associated with a lower likelihood of receiving guideline-concordant treatment recommendations and tumor board-concordant therapies: ECOG performance status, treatment intent, and cancer type. A significantly lower guideline and tumor board adherence was seen in patients with ECOG-PS ≥ 2 compared to patients with ECOG-PS < 2 (*P* < 0.05). With an increasing ECOG-PS, guideline and tumor board deviations increased (OR 3.64, 95% CI [1.70, 7.78], *P* = 0.001 resp. OR 3.15, 95% CI [1.54, 6.44], *P* = 0.002). Another independent factor was therapy intention. For patients with palliative intended therapy, logistic regression demonstrated a significantly lower guideline and tumor board adherence in contrast to patients receiving curative tumor therapy (OR 1.98, 95% CI [1.16, 3.38], *P* = 0.012 resp. OR 1.97, 95% CI [1.21, 3.23], *P* = 0.007). In addition, compared with colorectal cancer, patients with esophageal and gastric cancer had significantly higher guideline deviations (OR 3.23, 95% CI [1.38, 7.58], *P* = 0.007 resp. OR 5.95, 95% CI [3.13, 11.33], *P* < 0.001). Statistical analysis of tumor board adherence failed to yield significant results for the influencing factor of tumor entity (*P* = 0.063).

## Discussion

In this study, guideline adherence and implementation of tumor board recommendations for gastrointestinal cancer patients at a certified, tertiary referral academic oncological cancer center in Germany was assessed over a time-period of 1 year in each single presented case. To the best of our knowledge, this is the first study to investigate adherence to the German cancer treatment guidelines for patients with gastrointestinal tumors. To determine guideline adherence, we compared the given treatment recommendations of each MTB case with the associated guideline recommendations and categorized their adherence into complete adherence in 76%, minor deviations in 9%, and major deviations in 7% of all selected MTB cases. Previous studies determined guideline adherence based on NCCN guidelines and, in contrast to our study, by examining adherence to specific guideline recommendations among tumor therapies received (Bagante et al. 2019; Hamad et al. 2021; Jaap et al. 2018; Visser et al. 2012; Worhunsky et al. 2015; Zhao et al. 2018). In these studies, guideline adherence was only differentiated dichotomously into complete and non-adherence, designing a wide range. These studies likewise investigated patient- and tumor-specific factors associated with guideline deviations. Analogous to our results, these included higher comorbidity scores, higher tumor stage, and specific tumor entities (Chagpar et al. 2012; Hamad et al. 2021; Hines et al. 2015; Nishida et al. 2020). Compared to these other studies, our results showed no dependence of guideline adherence on patient age (Bagante et al. 2019; Boland et al. 2013; Chagpar et al. 2012; Hines et al. 2015; Kimmick et al. 2014; Nishida et al. 2020; Schiphorst et al. 2014; Visser et al. 2012; Worhunsky et al. 2015). Nevertheless, none of the studies has investigated, whether these patient and tumor factors were the actual reasons of guideline deviations (Hamad et al. 2021; Hines et al. 2015; Thiels et al. 2020). In our study, the most common cause of guideline deviation was the inclusion of patients into clinical trials. In accordance with the research focus of the UCCL, patients with gastric and esophageal cancer were often recommended therapies in the context of clinical trials; therefore, our study design resulted in many minor guideline deviations in these patients. Although tumor boards generally increase patients’ access to clinical trials (Kuroki et al. 2010; Mobley et al. 2020), these results may not be representative for MTBs of other hospitals.

Concerning the treatment implementation rate of 64% in our study, we compared our results with two previous studies investigating the tumor board adherence of gastrointestinal cancer patients. Tumor board adherence of colorectal carcinoma patients was studied by Wood et al. (2008), whereas Balzeby et al. (2006) focused on patients with tumors of the upper gastrointestinal tract. Both studies used a dichotomous, but not uniform or transparent definition of tumor board adherence, showing deviation rates of 10 and 15%. However, the specific causes of tumor board deviations were collected and both studies were consistent with our results, in which comorbidities, patient preferences, and tumor factors (e.g., increase in the extent of tumor disease, possible mutation detection, response to therapy) were identified as the most common reasons for deviating from tumor board recommendations.

In our analysis, comorbidities of cancer patients were one of the main influencing factors on guideline and tumor board adherence. 24% of guideline deviations were due to patient comorbidity status, and lower guideline adherence was significantly associated with an ECOG-PS ≥ 2 (*P* < 0.05). In accordance with the literature, it becomes clear that patients with higher comorbidity scores are less likely to be considered in guideline development, and, therefore, less likely to receive guideline-concordant treatment (Barth et al. 2016; Francke et al. 2008; Hahn et al. 2018; Stairmand et al. 2015; Vinod 2015). In medicine, guideline recommendations for patients with comorbidities are usually based on weak to moderate evidence or are not available at all (Lugtenberg et al. 2011). One of the reasons for this is the frequent exclusion of these patients from randomized controlled trials defining the new standards of care in oncology (Sedrak et al. 2021; Townsley et al. 2005).

Lack of information regarding patients’ comorbidities or wishes also occurs in tumor board meetings (Abukar et al. 2018; Bolle et al. 2019; Lamb et al. 2013; Wihl et al. 2021). In our study, patient comorbidities caused 24% of tumor board deviations and an ECOG-PS ≥ 2 was significantly associated with lower tumor board adherence (*P* < 0.05). Currently, there is neither a gold standard for comorbidity measurement in oncological patients nor a standard regarding patient-specific data that is required to make robust treatment recommendations in tumor boards (Sarfati 2012; Wihl et al. 2021). Furthermore, this may prevent MTB participants from making treatment decisions and from fully implementing them (Blazeby et al. 2006; Jalil et al. 2013; Wood et al. 2008).

Higher adherence rates could possibly be achieved by asking patients for specific treatment preferences before the MTB meeting takes place (Hollunder et al. 2018; Solomon et al. 2003). In our study, 16% of deviations from tumor board recommendations were due to divergent patient preferences. However, the perfect timing for consulting patients and asking for their preferences remains unclear. Especially considering that patient preferences might change during the course of treatment (Mallinger et al. 2006). The present study reflects that there are many situations in which tumor board adherence is not necessarily the optimal approach for patients and individual patient preference is ultimately decisive. To achieve the best outcome of patient care, the two paradigms of tumor- and patient-directed approach have to be combined.

Limitations of our study were the implementation as a single center study, focusing on gastrointestinal tumors alone, the newly implemented methodology for patient selection and adherence definition and the limited patient number. Therefore, further validation of our methodology and results are warranted. However, a major advantage of our study design is the individual patient-centered approach in combination with a systematic and objective quality assessment tool. Rather than examining the implementation of individual guideline recommendations in a cohort of patients, we looked at each individual case discussion to see whether evidence-based guideline recommendations were, first, available and, second, recommended by the respective MTB meetings. On this way we simultaneously subjected the guidelines to a review of the extent to which they are applicable in the local oncological setting. In this context the present study design allowed us to detect 6.4% of tumor board cases for which no specific guideline recommendations were available, especially by focusing not only on primary cases but all treatment lines. Therefore, our study could also contribute to new approaches of quality assurance for certified cancer centers.

## Conclusions

The assessment of guideline deviations and adherence to MTB decisions could become a meaningful and reproducible quality criterion for cancer centers in Germany and beyond. Individual patient care could be optimized by systematic measurement and comparison of guideline and tumor board adherences as well as causes of their deviation. Therefore, implementation of patient-reported outcomes and active patient involvement is warranted. Ultimately, the applicability of treatment guidelines could be enhanced using data from clinical care.

## ﻿Supplementary Information

Below is the link to the electronic supplementary material.

Supplementary file1 (DOCX 31 KB)

## Abbreviations

AWMF: Association of the Scientific Medical Societies in Germany
CRF: Case report form
DGHO: Deutsche Gesellschaft für Hämatologie und Medizinische Onkologie e.V.
EGJ: Esophagogastric Junction
GIST: Gastrointestinal stromal tumor
LAMN: Low-grade appendiceal mucinous neoplasm
MTB: Multidisciplinary tumor board
UCCL: University Cancer Center Leipzig
UKL: University Hospital Leipzig
