# Supplementary material for: Guideline adherence and implementation of tumor board therapy recommendations for patients with gastrointestinal cancer
Source: J Cancer Res Clin Oncol. 2022 Apr 8;149(3):1231–40. doi: 10.1007/s00432-022-03991-6 (PMC9984328; doi:10.1007/s00432-022-03991-6)
Supplement: Supplementary file 1 — Supplementary file1 (DOCX 31 KB) [file 432_2022_3991_MOESM1_ESM.docx]

**Supplemental Table 1**

|  | Patient 1 (Example of Guideline deviation) | Patient 2 (Example of Tumor board deviation) |
| --- | --- | --- |
| MTB Data: | | |
| Patient characteristics | 79 years old male patient, ECOG 2 | 55 years old female patient, ECOG 0 |
| Tumor characteristics | Squamous cell carcinoma of the esophagus  UICC Stage III, TNM: cT2 N2 M0, G2  no previous therapies | Neuroendocrine tumor of the pancreas  UICC Stage IV, TNM: cT4, N2, M1 (PUL), G3, Ki67 30-40%  no previous therapies |
| MTB recommendation | Primary radiotherapy due to limited general condition | Palliative chemotherapy with Streptozotocin + 5-Fluorouracil |
| Major criteria | Radiotherapy | Chemotherapy |
| Minor criteria | - | Chemotherapeutics: Streptozotocin + 5-Fluorouracil |
| Guideline adherence: | | |
| Guideline recommendation | Definitive radiochemotherapy should be given regardless of the histologic entity of the esophageal cancer if the tumor is deemed surgically/ endoscopically unresectable at an interdisciplinary tumor conference or if a patient is functionally inoperable or refuses surgery after extensive informed consent.  (Leitlinienprogramm Onkologie (Deutsche Krebsgesellschaft, Deutsche Krebshilfe, AWMF) 2018) | Chemotherapy should be used as first-line therapy in patients with pancreatic NET with high hepatic tumor burden (>25%), significant tumor progression in 6 months, or G2 NET with >10% Ki-67. The combination of Streptozotocin and 5-Fluorouracil should be used as standard chemotherapy for pancreatic NET. Capecitabine/ Temozolomide can also be given as an alternative oral chemotherapy regimen for pancreatic NET.  (Deutsche Gesellschaft für Gastroenterologie, Verdauungs- und Stoffwechselkrankheiten (DGVS) et al. 2018) |
| Major criteria | Radiotherapy and Chemotherapy | Chemotherapy |
| Minor criteria | - | Chemotherapeutics: Streptozotocin + 5-Fluorouracil or Capecitabine + Temozolomide |
| Guideline adherence | **Major guideline deviation**  → because of a deviation in at least one major criterion (chemotherapy) between the MTB and guideline recommendations | **Complete guideline adherence**  → because of concordance in all major and minor criteria between the MTB and guideline recommendations |
| Causes of deviation | Comorbidities (ECOG 2) | - |
| Tumor board adherence: | | |
| Course of treatment  (≤ 3 months) | Performance of intensity-modulated 7 MV photon teletherapy (9/63) of the esophageal tumor, affected lymph nodes, and adjacent para-esophageal lymph drainage areas with simultaneous integrated boost in the esophageal tumor and LKM area; total dose 63 Gy | Due to impaired renal function with necessary dose reduction, implementation of oral chemotherapy with Capecitabine + Temozolomide. |
| Major criteria | Radiotherapy | Chemotherapy |
| Minor criteria | Radiation dose: 63 Gy with integrated boost, localization: primary tumor + local lymph nodes + lymph drainage areas | Chemotherapeutics: Capecitabine + Temozolomide |
| Tumor board adherence | **Complete tumor board adherence**  → because of concordance in all major and minor criteria between the MTB recommendation and the patient’s course of treatment | **Minor tumor board deviation** → because of deviation in at least one minor criteria (chemo-therapeutics) between the MTB recommendation and the patient’s course of treatment |
| Causes of deviation | - | Comorbidities (renal insufficiency) |
